# Cooking Alters the Metabolites of Onions and Their Ability to Protect Nerve Cells from Lead Damage

**DOI:** 10.3390/foods13223707

**Published:** 2024-11-20

**Authors:** Li Zhao, Liping Wang, Nan Wang, Xinchang Gao, Bin Zhang, Yufen Zhao, Ning Wang

**Affiliations:** 1 Institute of Drug Discovery Technology, Ningbo University, Ningbo 315211, China; 2111072066@nbu.edu.cn (L.Z.); 2311130047@nbu.edu.cn (L.W.); gxch43@126.com (X.G.); 2 College of Food Sciences and Engineering, Ningbo University, Ningbo 315211, China; 2415390309@nbu.edu.cn; 3 Health Science Center, Ningbo University, Ningbo 315211, China; zhangbin1@nbu.edu.cn; 4 Qian Xuesen Collaborative Research Center of Astrochemistry and Space Life Sciences, Ningbo University, Ningbo 315211, China; 5 Key Lab of Bioorganic Phosphorus Chemistry & Chemical Biology, Department of Chemistry, Tsinghua University, Beijing 100084, China

**Keywords:** *Allium cepa* L., cooking, metabolomics, lead exposure, antioxidation, chelation

## Abstract

Onions (*Allium cepa* L.) are nutritious vegetables; however, variations in processing methods can influence their chemical composition and functional properties. Raw processing and cooking are the two main food-processing methods for onions, but it is not clear what kind of changes these two methods cause. In the present study, ultrahigh-resolution liquid chromatography–mass spectrometry (UHPLC–MS) was utilized to observe the changes in onion composition during cooking and to investigate the protective effects of raw and cooked onion extracts against lead damage in vitro and at the cellular level. Many compounds were identified, including amino acids, nucleosides, flavonoids, and organosulfur compounds. Cooking causes changes in the content of numerous amino acids (e.g., DL-glutamine) in onions and increases nucleoside content (e.g., 5’-S-methyl-5’-thioadenosine, adenine). Both raw and cooked onion extracts can reduce neuronal cell damage caused by lead exposure, but cooking increased the free radical scavenging (e.g., DPPH, ABTS, hydroxyl radicals) and chelating of lead ions (up to about 25%) of the onion extracts. In conclusion, cooking can cause changes in the chemical composition of onions and increase their antioxidant and lead chelating capacity.

## 1. Introduction

Onions (*Allium cepa* L.) are perennial herbs of the *Liliaceae* and *Allium* family [1]. They are one of the most essential vegetables and are cultivated in diverse climatic conditions worldwide. Historically, onions have been widely used in different modes as a food and spice in many dishes in almost all cultural areas. Not only are they famous for their unique flavor and aroma, but they are also a rich source of nutrition. The complex and diverse nutrients in onions have been studied extensively. Phenolic acids, flavonoids (quercetin and kaempferol), essential amino acids, anthocyanins, and organosulfur compounds (allicin) [2,3] have been identified. Onions have been used in traditional medicine to treat and prevent various diseases for many years, such as coronary heart disease, gastrointestinal disorders, different types of cancer, and inflammatory diseases [2]. It is well established that food can be consumed in a multitude of ways, and various processing methods can elicit distinct changes in its properties. For instance, thermal treatment facilitates the release of volatile organosulfur compounds from plants such as chili peppers and garlic [4]. In China, raw preparation and cooking are the predominant methods of processing onions, in contrast to the deep-frying techniques commonly employed in Europe and the United States. However, whether cooking affects the nutrients in onions and their biological functions has not been studied.

Lead is a long-lasting inorganic environmental pollutant that has global effects on humans and animals. Lead can enter the human body via various routes, including inhaling lead fumes and dust from industrial smelting processes or ingesting food and water contaminated with lead [5]. Inhaled or ingested lead can accumulate in various tissues, such as the brain, liver, and kidneys. This accumulation can cause cellular damage through mechanisms such as oxidative stress, ultimately leading to impaired bodily functions [6]. Previously, lead detoxification primarily relied on chelating agents [7]. However, these agents only reduce the body’s lead levels and do not repair the damage caused by lead exposure. This reality has led to the search for safer, more reliable lead-eliminating substances. Numerous studies have demonstrated that organosulfur compounds (e.g., allicin) [8] and flavonoids (e.g., tea catechins) [9,10] possess the ability to attenuate the deleterious effects associated with lead exposure. Onions are rich in flavonoids and organosulfur compounds, all of which have a lead-removing effect [10,11]. This study explored the antioxidant attributes, chelation capabilities, and protective efficacy of both raw and cooked onion-derived external extracts, specifically regarding their role in countering lead-induced neurotoxicity in cellular models.

## 2. Materials and Methods

### 2.1. Plant Materials

Common fresh purple onions (*Allium cepa* L.) were purchased from the local market. The onion skin and root were removed, and the onion was chopped into small pieces and homogenized. A part of the onion homogenate (without additional water) was cooked in a pressure cooker at 121 °C for 20 min. After cooking, it was placed in an ice water bath to cool down quickly. Then, the raw or cooked onion homogenate samples were lyophilized for standby using a lyophilizator (Biocool, Beijing, China). The lyophilized samples were stored at −80 °C until use.

### 2.2. Extraction Method

The extraction of raw and cooked onions was optimized according to previous studies [3,12]. Approximately 0.1 g of lyophilized raw or cooked onion homogenate was mixed with a solvent (deionized water: methanol = 20:80, *v*/*v*) in proportion (30 mg/200 μL), and the mixture was shaken and sonicated for 10 min and then centrifuged at 1000 rpm for 10 min. The supernatant was collected, spun, and dried by adding 20% methanol, twice the volume of the supernatant. The extract was filtered using a 0.22 μm aqueous phase membrane, and the filtrate was used for ultrahigh-resolution liquid chromatograph–mass spectrometer (UHPLC–MS, Thermo Scientific^TM^ U3000–Thermo Scientific^TM^ Q Exactive Plus^TM^, Thermo Fisher, Waltham, MA, USA) analysis.

### 2.3. UHPLC–MS Analysis

1 mL of filtrate was added to an injection vial and analyzed using UHPLC-MS (Thermo Scientific^TM^ Q Exactive Plus^TM^, Thermo Fisher, Waltham, MA, USA). Ten parallel groups were set up for each sample. Chromatographic conditions: The separation of compounds from both raw and cooked onion extracts was performed on a Waters ACQUITY UPLC BEH C18 column (1.7 μm, 2.1 mm × 150 mm; Waters, Milford, MA, USA) maintained at 30 °C. In the positive ion mode, two mobile phases were used: A, consisting of 5% acetonitrile in 95% deionized water with 0.1% formic acid; and B, acetonitrile. For the negative ion mode, the mobile phases were: A, 0.3% aqueous ammonia solution; and B, acetonitrile. The gradient elution program for the samples was as follows: 0–5 min, 2% B; 5–25 min, 2–30% B; 25–35 min, 30–70% B; 35–45 min, 70–90% B; 45–50 min, 90% B; 50–51 min, 90–2% B; and 51–60 min, 2% B.

The column eluate directly entered the mass spectrometer fitted with a heated electrospray ionization probe. The instrument was operated in the positive ionization mode to detect compounds. Full MS-ddMS (2 TOP5) spectra were recorded in the *m*/*z* range from 85 to 1000 with a resolution of 70,000 Hz. The parameters used were as follows: sheath gas, 40 Arb; auxiliary gas, 10 Arb; reverse blowing, 0 Arb; positive spectrum spray voltage, 3.6 kV; capillary temperature, 320 °C; and auxiliary gas temperature, 350 °C.

### 2.4. Cell Proliferation Assay

Rat adrenal pheochromocytoma PC12 cells, human glioblastoma U343MG cells, U251MG cells, and human hepatoma cell line Hep G2 were purchased from the National Collection of Authenticated Cell Cultures (Shanghai, China).

Cell viability was determined using the Cell Counting Kit–8 (CCK–8), which was modified based on the method of Jiang et al. [13]. Cells were treated with different concentrations of samples and lead acetate solutions for 24 and 48 h, and then CCK-8 staining was performed for 2 h. Absorbance was measured at a wavelength of 450 nm using the SpectraMax paradigm multimode detection platform (Molecular Devices, San Jose, CA, USA).

### 2.5. Reactive Oxygen Species (ROS) Assay

The U251MG cells were damaged with 10 µmol/L lead(II) ions solution for 24 h and then treated with onion extracts for another 24 h. A dichlorofluorescin diacetate (DCFH–DA) probe was used to determine the level of ROS in cells [14]. The probe was loaded using a serum-free medium and incubated for 20 min at 37 °C in a cell incubator. Next, the cells were collected. Fluorescence intensity was measured using a 488 nm excitation wavelength and 525 nm emission wavelength.

### 2.6. Antioxidant Activity Assays

#### 2.6.1. 1,1-Diphenyl-2-picrylhydrazyl (DPPH) Radical Scavenging Assay

The DPPH radical scavenging capacity was determined using 96-well microtiter plates (Thermo Fisher Scientific, Waltham, MA, USA), modified from the method of Jing et al. [15]. Then, 40 μL of the sample was mixed with 160 μL of DPPH working solution (0.2 mmol/L) and incubated for 30 min at 23–25 °C in the dark. At least three parallel experiments were performed for each sample. The absorbance value was measured at 517 nm. The radical scavenging capacity was calculated using Formula (1):

(1) $$Radical\;scavenging\;rate\;\left(\%\right)=\left(1-\frac{A_{1}-A_{2}}{A_{3}}\right)\times 100\%\;$$

A_1_ in the equation refers to the absorbance values of the sample mixture and the radical working solution. A_2_ is the absorbance of the sample at different concentrations (with blank solvent). A_3_ is the absorbance of the control (radical solution without sample).

#### 2.6.2. 2,2′-Azino-bis(3-ethylbenzothiazoline-6-sulfonic Acid) (ABTS) Radical Scavenging Assay

The ABTS assay was based on the method of Jing et al. [15], with modifications. ABTS radical cation solution was prepared by reacting 7 mmol/L ABTS solution and 2.45 mmol/L potassium persulfate solution (1:1 *v*/*v*) at 23–25 °C overnight in the dark. Before the assays, the solution was diluted with 80% methanol to achieve an absorbance value of 0.7 ± 0.02 at 734 nm. Then, 180 μL of ABTS radical cation solution was reacted with 15 μL of sample solution for 6 min, and the absorbance was recorded at 734 nm. Each sample was taken in at least thrice. The scavenging ability of ABTS was calculated as described in Section 2.6.1.

#### 2.6.3. Hydroxyl Radical Scavenging Assay

The experiment was conducted by modifying the method of Hong et al. [16]. Here, 20 μL of the sample, 20 μL of 6 mmol/L FeSO_4_ solution, and 20 μL of 6 mmol/L H_2_O_2_ solution were reacted for 10 min. Subsequently, 60 μL of 6 mmol/L salicylic acid–ethanol solution was added, and the absorbance value was measured at 510 nm after 30 min in a 37 °C water bath. Ethanol was used instead of salicylic acid in the control group, and 80% methanol was used instead of the blank sample. All experiments were repeated at least thrice. The hydroxyl radical scavenging rate can be calculated using the formula provided in Section 2.6.1.

#### 2.6.4. Superoxide Anion Radical Scavenging Assay

This experiment was performed by modifying the method of Hong et al. [16]. Here, 40 μL of the sample was mixed with 120 μL of Tris–HCl (50 mmol/L, pH 8.2) and left in the dark for 20 min. Subsequently, 16 μL of 5 mmol/L pyrogallol solution was added, and the mixture was left for 4 min. The reaction was stopped by adding 1.5 mol/L HCl. Finally, the absorbance of the solution was measured at 325 nm. The formula mentioned in Section 2.6.1 was used to determine the scavenging ability of superoxide anion radicals.

### 2.7. Lead(II) Ion Chelation Assay

We used the experimental principle of Low et al. [17] and Yuejuan and Daozong [18] to determine the ability of onion extracts to chelate lead ions. The working solution consisted of a sample, deionized water, and 0.5 mg/mL lead(II) ions solution (2:5:2, *v*/*v*/*v*), and they were reacted at 37 °C for 30 min. Then, 200 μL of dithizone (0.04 mg/mL), 500 μL of NH_4_Cl–NH_3_·H_2_O (10 mg/mL), 650 μL of 4-(2-pyridylazo)resorcinol (PAR) (0.05 mg/mL), 200 μL of 1,10-phenanthroline monohydrate (60 mg/mL), 200 μL of ammonium thiocyanate solution (10 mg/mL), 200 μL of potassium sodium tartrate tetrahydrate (0.5 mg/mL), 200 μL of working solution, and 350 μL of deionized water were reacted in a reaction vessel, and the absorbance value of the reaction solution was measured at 530 nm. All experiments were repeated at least thrice. Chelating capacity was calculated using Formula (2):

(2) $$The\;chelating\;ability\;\left(\%\right)=\left(\frac{A_{control}-A_{sample}}{A_{control}}\right)\times 100\%$$

Deionized water was used instead of the sample solution as the working solution in the control group. EDTA was used as a positive control instead of the sample solution.

### 2.8. Statistical Analysis

For the metabolomics experiments, samples were randomly selected, with 10 replicates per group. Statistical analyses, including both multivariate and univariate statistical analyses, were conducted. The specific methods employed were partial least squares discriminant analysis (PLS–DA) and hierarchical cluster analysis (HCA), which were used to examine the changes in the chemical composition of onions. PLS–DA, score plots of variable importance in projection (VIP), and heatmap were generated using MetaboAnalyst v. 6.0 (https://www.metaboanalyst.ca/) (accessed 10 October 2024). Volcano plots were generated using R. v. 4.2.2 (https://www.r-project.org) (accessed 12 October 2024). Subsequent cell experiments were performed in at least triplicate. The specified number of replicates is shown in the Results. Analysis of variance was performed using GraphPad Prism 9.0.0 to assess for significant differences. One-way or two-way ANOVA was performed. *p*-values < 0.05 were considered statistically significant.

## 3. Results

### 3.1. Effect of Cooking on Onion Metabolomics

The compounds of raw onions (R–onion) and cooked onions (H–onion) were analyzed using UHPLC–MS.

After filtering out the blank background, a total of 13,347 positive ion signals and 3319 negative ion signals were obtained through the database search in Compound Discoverer 3.2. Following the filtering and qualitative comparison, 3136 positive ion signals and 1162 negative ion signals were successfully annotated within the existing databases of Compound Discoverer 3.2, which include mzCloud, ChemSpider, mzVault, and Mass List. Additionally, by setting the best match score threshold to over 80%, 142 compounds were identified in positive mode from the mzCloud database. In negative mode, 59 compounds were similarly identified with the same match score threshold.

#### 3.1.1. Partial Least Squares Discriminant Analysis (PLS–DA)

**Figure 1:**
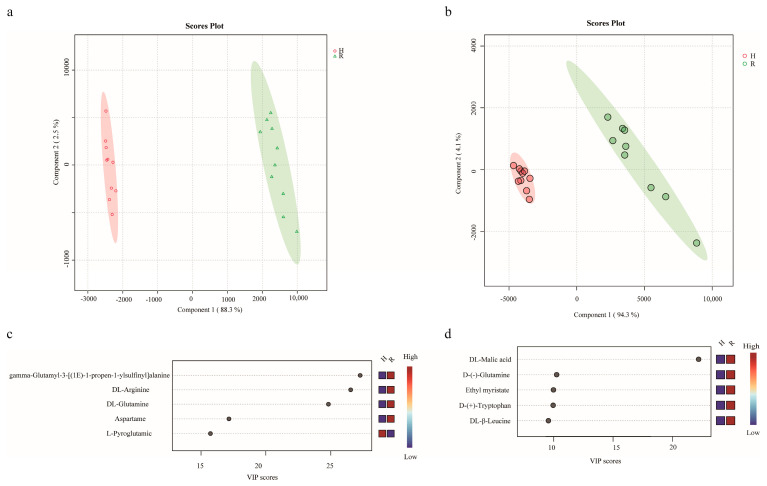
Partial least squares discriminant analysis. (**a**) The 2D scores plot of PLS–DA in positive modes. (**b**) The 2D scores plot of PLS–DA in negative modes. (**c**) Score plots of VIP in positive modes. (**d**) Score plots of VIP in negative modes.

The compound information, including compound names and peak areas obtained from Compound Discoverer 3.2, was imported into MetaboAnalyst v. 6.0 (https://www.metaboanalyst.ca/) (accessed 10 October 2024). PLS–DA was then employed to comprehensively analyze and evaluate the metabolomic data from both raw and cooked onions (Figure 1a,b). The two most influential principal components were observed in PLS–DA (Figure 1a), which describes 88.3% and 2.5% of the cumulative contribution rate in positive modes. This indicates that the data from the UHPLC–MS analysis have a good display on the two principal components, and Component 1 and Component 2 can reflect the main characteristic information of the samples. The PLS–DA model in negative mode exhibited two primary clusters, similar to the findings in positive mode. Component 1 accounted for a larger proportion of the variance (94.3%), while Component 2 explained a smaller proportion (4.1%) (Figure 1b). In the experiment, samples of raw and cooked onions were clustered in the left and right parts of the figure, indicating that the differences between the two types of onions were significant. VIP is an essential metric in PLS–DA (Figure 1c,d). The VIP based on the PLS–DA model was estimated to determine the key variables driving the separation of raw and cooked onions. The colored boxes on the right indicate the relative concentrations of the corresponding metabolites in each study group. The higher the value of VIP for a variable is, the more critical it is in distinguishing between raw and cooked onions. Variables with VIP values greater than one are considered to be particularly essential to the model. Based on the VIP scores, the following metabolites were identified as the most significant contributors to the separation of raw and cooked onions in positive mode: gamma-glutamyl-3-[(1E)-1-propen-1-ylsulfinyl]alanine, DL-arginine, DL-glutamine, aspartame, and L-pyroglutamic acid. In negative mode, the key metabolites were DL-malic acid, D-(-)-glutamine, ethyl myristate, D-(+)-tryptophan, and DL-β-leucine.

#### 3.1.2. Analysis of Differential Compounds

**Figure 2:**
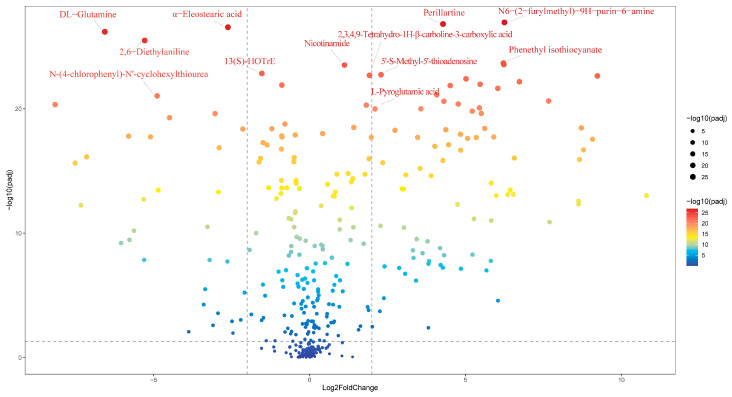
Volcanic diagram of the difference between raw and cooked onions.

Volcano plots serve as an intuitive tool when focusing on differential components. As illustrated in Figure 2, the volcano plot was generated by first screening for matches with a greater than 60% match score in mzCloud, followed by setting the ratio = cooked onion/raw onion (H/R), *p*-value = 0.001, and Log2 fold change = 2. The component with the largest and statistically significant variance among the samples was N6-(2-furylmethyl)-9H-purin-6-amine. As shown in Figure 2, N6-(2-furylmethyl)-9H-purin-6-amine appears in the top right of the volcano plot. The content of N6-(2-furylmethyl)-9H-purin-6-amine increased to approximately 75 times that of raw onions after cooking. N6-(2-Furylmethyl)-9H-purin-6-amine, also known as kinetin, is the first stable secondary DNA damage product identified [19]. Miller et al. first discovered kinetin in high-pressure sterilized DNA samples from herring sperm in 1955 [20,21] under conditions similar to those in our experiments. During cooking, onions experience extreme conditions of heat and pressure, which stimulate oxidative stress in cells [22]. Oxidative stress causes DNA to fragment [23,24]. This may account for the higher levels of N6-(2-furylmethyl)-9H-purin-6-amine found in cooked onions.

It is worth mentioning that α-eleostearic acid (ratio: H/R = 0.16), DL-glutamine (ratio: H/R = 0.01), 2,6-diethylaniline (ratio: H/R = 0.025), and 13(S)-hydroxyoctadecatrienoic acid (13(S)-HOTrE) (ratio: H/R = 0.342) caught our attention because their contents were significantly reduced after cooking. α-eleostearic acid is a highly unsaturated fatty acid. A study by Talita A. Comunian et al. on the stability of pomegranate seed oil found that heating methods significantly decrease the concentration of α-eleostearic acid by the end of storage [25]. This observation aligns with our experimental results. 13(S)-HOTrE, also a member of the fatty acid family, exhibited a similar trend of reduction after cooking. This finding is consistent with the observations made by Huijie Wei [26] et al. regarding the decrease in 13(S)-HOTrE in baked tea. 2,6-diethylaniline, a metabolite of alachlor (a type of herbicide) in biological systems, exhibits systemic toxicity [27]. The significant reduction in its content after cooking implies that the cooking process can mitigate the adverse health effects associated with herbicide use during plant cultivation. Interestingly, the content of DL-glutamine in onions decreases significantly after cooking, while the level of L-pyroglutamic acid increases. Previous studies have demonstrated that DL-glutamine can undergo cyclization under high temperature and pressure conditions, converting to pyroglutamic acid [28], corroborating our experimental observations.

Perillartine, with a ratio of H/R = 19.114, is a natural sweetener that has been detected in various plants and herbal preparations, including *Lonicera japonica* Flos and the herb pair *Gastrodia elata* Blume and *Acorus tatarinowii* Schott [29,30]. Omayma Bouzekr [31] et al. reported that perillaldehyde can be converted to perillartine under heating conditions during their study of the essential oil components of *Ammodaucus leucotrichus* Coss. Phenethyl isothiocyanate (ratio: H/R = 73.32) can be used as a flavoring agent to impart a pungent taste to foods and is commonly found in condiments such as mustard and horseradish. It is a degradation product of glucosinolates, and processing and cooking lead to the breakdown of glucosinolates and the formation of volatile compounds [32]. This suggests that the increase in phenethyl isothiocyanate content in onions after cooking is a reasonable outcome. Nicotinamide, the amide form of niacin, remains stable under short-term heating. Kondjoyan A. et al. [33] observed that during the cooking of beef, there is a reduction in niacin levels, while nicotinamide levels remain relatively stable. Research has shown that nicotinamide is more bioavailable than niacin [34], suggesting that cooking can bring about positive changes in onions, making them more suitable for human consumption. 5’-S-Methyl-5’-thioadenosine (ratio: H/R = 4.822) has been detected in higher concentrations in *Sinojackia xylocarpa* Hu seeds under high-temperature storage conditions [35]. This finding is consistent with our experimental observations. 5’-S-Methyl-5’-thioadenosine is a nucleoside that can be reversibly converted from adenine and 5-(methylthio)-ribose-1-phosphate (MTRP) through the action of specific enzymes [36]. Furthermore, under high-temperature conditions, DNA can fragment into various products [37], which may account for the observed increase in 5’-S-Methyl-5’-thioadenosine levels. 2,3,4,9-Tetrahydro-1H-β-carboline-3-carboxylic acid (ratio: H/R = 3.743) has been detected in *Lepidium meyenii* (Maca) [38]. This compound is a heterocyclic amine that can be formed via the Maillard reaction involving amino acids, creatine, and reducing sugars [39]. Tryptophan Amadori rearrangement products can react with carbonyl compounds, such as acetaldehyde or α-ketonic acids, to produce 2,3,4,9-tetrahydro-1H-β-carboline-3-carboxylic acid and 1-methyl-1,2,3,4-tetrahydro-β-carboline-3-carboxylic acid via the Pictet-Spengler pathway. These compounds are then oxidized and decarboxylated to generate norharman and harman, respectively. In a study by Xuefei Li et al. [40] on fried chicken leg meat, higher levels of 2,3,4,9-tetrahydro-1H-β-carboline-3-carboxylic acid were detected in the chicken legs after high-temperature frying. This finding is consistent with our detection results.

#### 3.1.3. Cluster Analysis of Raw and Cooked Onions

In total, 3135 positive ion signals and 1162 negative ion signals were successfully annotated using the existing databases in Compound Discoverer 3.2 (including mzCloud, ChemSpider, mzVault, and Mass List). The analysis of these compounds revealed that out of the 3135 positive ion signals, 507 were identified as organic sulfur compounds (such as alliin and allicin). Among the 1162 negative ion signals, 177 were identified as organic sulfur compounds (such as S-phenyl-D-cysteine and diallylheptasulfane). Information regarding these compounds is given in Tables S1 and S2.

After categorizing and organizing the compound information, 143 compounds detected in the positive ion mode had a match score greater than 80% in the mzCloud database. These compounds can be classified into several categories: alkaloids (e.g., nicotine and trigonelline), amino acids and derivatives (e.g., DL-glutamine and DL-arginine), flavonoids (e.g., quercetin, kaempferol, and luteolin-3’,7-diglucoside), lipids (e.g., 13(S)-HOTrE and (12Z)-9,10,11-trihydroxyoctadec-12-enoic acid), nucleotides and derivatives (e.g., cytarabine and 5’-S-methyl-5’-thioadenosine), phenols (e.g., cyanidin and phloroglucinol), and other compounds (e.g., cafestol and 4-ethynylaniline). In the negative ion mode, 59 compounds had a match score greater than 80% in the mzCloud database. These compounds primarily belong to the following categories: amino acids and derivatives (e.g., L-histidine and DL-β-leucine), flavonoids (e.g., astragalin), lipids (e.g., α-eleostearic acid and oleic acid), nucleotides and derivatives (e.g., guanosine), and other compounds (e.g., 1-naphthoic acid). Information regarding these compounds is given in Tables S3 and S4.

**Figure 3:**
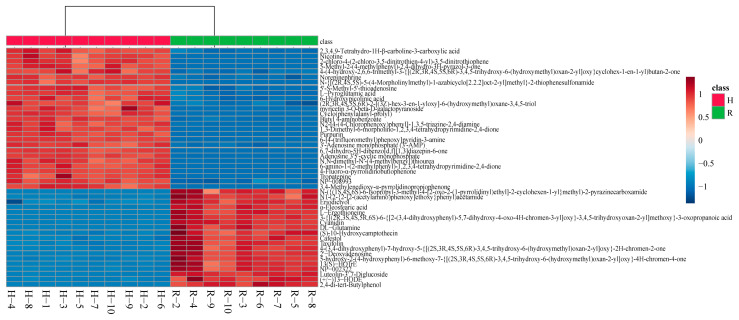
Hierarchical clustering analysis of raw and cooked onions.

After consolidating and organizing the compounds, a heatmap was generated using the compounds that showed significant differences, as depicted in Figure 3. The heatmap clearly illustrates distinct differences between the sample groups, with no significant variations observed within each group. The levels of certain nucleotides and their analogs, such as 5’-S-methyl-5’-thioadenosine and adenine 3’,5’-cyclic monophosphate, were found to be higher in cooked onion samples compared to raw onion samples. This increase can be attributed to the fragmentation of onion DNA into various nucleotides and their analogs under high-temperature and high-pressure conditions [22,24,37]. Flavonoids have been the subject of extensive research, and in this study, numerous flavonoids were detected in both positive and negative ion modes. An interesting observation was the presence of various flavonoid glycosides, such as myricetin 3-O-β-D-galactopyranoside and hyperoside. Studies have shown that flavonoid glycosides generally have higher bioavailability in the human body compared to their aglycone forms [41,42,43,44,45]. Notably, quercetin glycosides, which are among the most bioavailable, are found in onions. A significant increase in the content of myricetin 3-O-β-D-galactopyranoside was observed after cooking, suggesting that cooking onions enhances the bioavailability of flavonoids, thereby making them more beneficial to human health.

### 3.2. Protective Effect of Onions on Lead Damage in Nerve Cells

#### 3.2.1. Cell Modeling and Cytotoxicity

**Figure 4:**
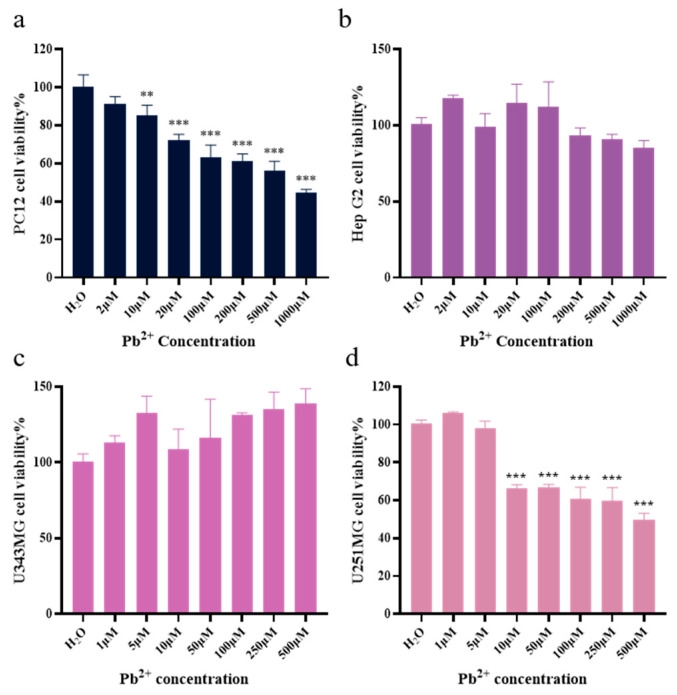
Cell viability under lead(II) exposure. (**a**) Cell viability of PC12 cells. (**b**) Cell viability of Hep G2 cells. (**c**) Cell viability of U343MG cells. (**d**) Cell viability of U251MG cells. ** *p* < 0.01, *** *p* < 0.005. The results are shown as the mean ± SD (*n* = 6).

Cell proliferation is an essential feature of living organisms, and cell viability is an essential factor in evaluating the level of cell proliferation [46]. In the present study, cell viability was evaluated using the CCK–8 method. First, cell models were determined by screening cell and lead(II) ions modeling concentrations, followed by cell viability assessment to further determine the toxicity ranges of the individual samples and monomer concentrations. As shown in Figure 4a–d, the results indicate that PC12 cells (IC_50_ = 80.77 μM) and U251MG cells (IC_50_ = 6.924 μM) were sensitive to lead ions. By contrast, Hep G2 cells and U343MG cells were not sensitive to lead(II).

**Figure 5:**
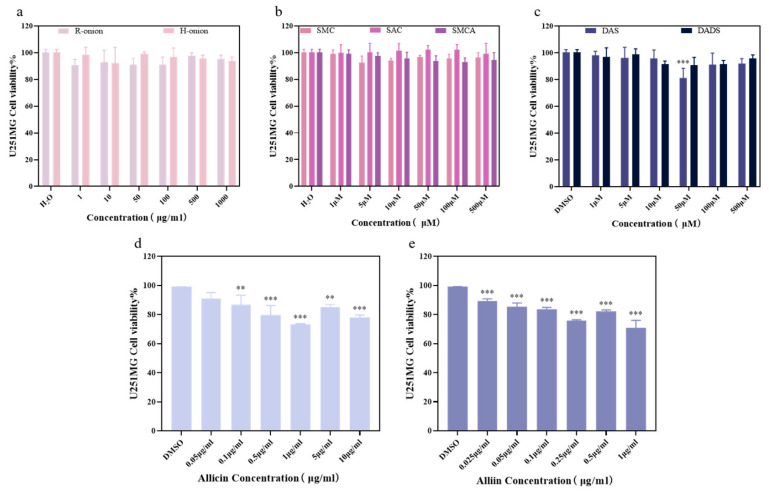
Cell viability. (**a**) Cell viability of U251MG cells treated with raw and cooked onion extracts for 48 h. (**b**) Cell viability of U251MG cells treated with SMC, SAC, and SMCA for 48 h. (**c**) Cell viability of U251MG cells treated with DAS and DADS for 48 h. (**d**) Cell viability of U251MG cells treated with allicin for 48 h. (**e**) Cell viability of U251MG cells treated with alliin for 48 h. ** *p* < 0.01, *** *p* < 0.005. The results are shown as the mean ± SD (*n* = 6).

After measuring the concentration of lead(II) ions, experiments were performed to determine the toxicity of onion extracts and compounds in PC12 cells and U251MG cells. Experimental results for PC12 cells are shown in Figure S1. Figure 5 shows the experimental results that onion samples (both raw and cooked onion samples) were not cytotoxic to U251MG cells (concentration range: 1–1000 μg/mL). Similarly, S-methyl-L-cysteine (SMC), S-allyl-L-cysteine (SAC), and S-methyl-L-cysteine sulfoxide (SMCA) were nontoxic to U251MG cells and could be used for subsequent experiments. Interestingly, diallyl disulfide (DADS) was nontoxic within the experimental concentrations, whereas diallyl sulfide (DAS) showed significant toxicity at 50 μM relative to its DMSO solvent group. Allicin and alliin showed significant toxicity to U251MG cells within the concentration range of the experimental setup and were not suitable for further use in subsequent experiments. Therefore, we selected raw onion extracts, cooked onion extracts, SMC, SAC, SMCA, DAS, and DADS to be used in the subsequent experiments in neuronal cells and to investigate the mechanism of action.

#### 3.2.2. Protective Effect of Onion Extracts on Neuronal Cell Damage Under Lead(II) Exposure

**Figure 6:**
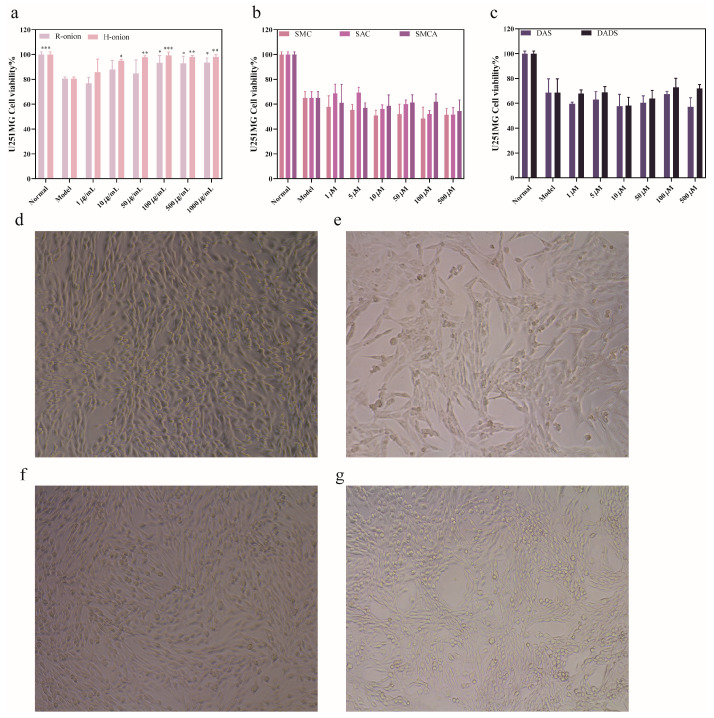
Cell viability and cell morphology. (**a**) Cell viability of 10 μM lead(II)-injured cells for 24 h, followed by treatment of cells with fresh and cooked onion extracts for 24 h. (**b**) Cell viability of 10 μM lead(II)-injured cells for 24 h, followed by treatment of cells with SMC, SAC, and SMCA for 24 h. (**c**) Cell viability of 10 μM lead(II)-injured cells for 24 h, followed by treatment of cells with DAS and DADS for 24 h. (**d**) Cell morphology of U251MG cells in the normal group. (**e**) Cell morphology of U251MG cells in the model group. (**f**) Cell morphology of U251MG cells in the 1000 μg/mL cooked onion group. (**g**) Cell morphology of U251MG cells in the 1000 μg/mL raw onion group. The total duration of the experiment was 48 h. * *p* < 0.05, ** *p* < 0.01, *** *p* < 0.005. The results are shown as the mean ± SD (*n* = 9).

The protective effect of onion extract on neuronal cell damage under lead(II) exposure was explored by CCK–8 assay. As shown in Figure 6, the cell viability of U251MG cells treated with 10 μM lead ions was significantly decreased, indicating successful modeling. The raw onion extract samples significantly enhanced the cell viability of U251MG cells at high (1000 and 500 μg/mL) and medium (100 μg/mL) concentrations, which suggests that raw onion extracts can protect nerve cells at higher concentrations. It was unexpected to discover that the extract of cooked onions not only improves cell viability at high concentrations (1000 and 500 μg/mL) and medium concentrations (100 and 50 μg/mL) but also significantly boosts cell viability of U251 cells at low concentrations like 10 μg/mL (Figure 6a). The results of the PC12 cell experiments are shown in Figure S2. The results were similar to those of the U251MG cell experiments in that the onion extract attenuated the damage of lead ions to PC12 cells at high concentrations. In addition, from the cellular morphology, we observed that the cells in the model group exhibited a reduced number of cells and wrinkled cytosol compared with the cells in the normal group, which had full cytosol with elongated and pronounced cellular dendrites. By contrast, the cells treated with onion extracts showed an increase in cell number, and the wrinkled cytosol was reproduced to some extent to become full. This further suggests that onion extracts protect against lead(II)-induced U251MG cell damage. In response to the comparison of the protective effects between raw and cooked onions, we found that cooked onions showed some advantages, but these advantages were not significant, and there was no statistical difference in the protective effects of the two. Unfortunately, the nontoxic sulfur-containing compounds in the experiment did not show the effect of improving cell viability. This suggests that the substances in onions that play a lead-eliminating role are not those in the experiment and that the specific substances that play a role need to be further investigated.

#### 3.2.3. Antioxidant Capacity of Onion Extracts

Many studies have shown that lead(II) ions cause damage to the body mainly through oxidative stress [47,48]. In metabolomics assays, we have identified several antioxidant components (e.g., quercetin and quercetin-3β-D-glucoside) in onions. We hypothesized that onion extracts protect nerve cells by acting as an antioxidant. To confirm our hypothesis, we conducted in vitro tests to measure the ability of onion extracts to scavenge free radicals and reduce reactive oxygen species at the cellular level.

**Figure 7:**
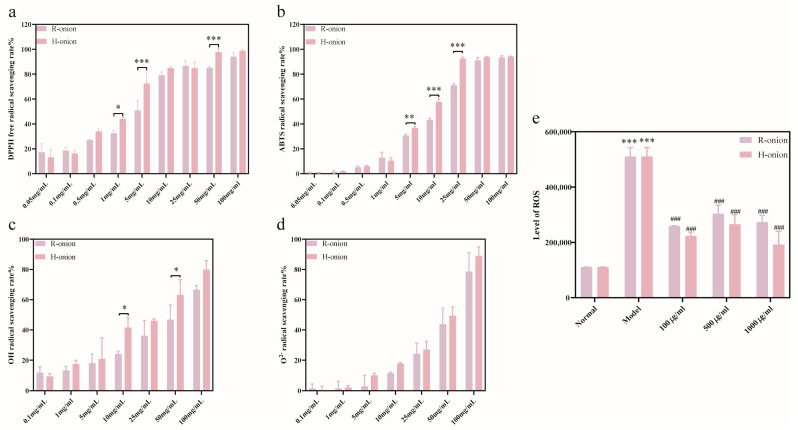
Antioxidant capacity of onions. (**a**) DPPH free radical scavenging capacity. (**b**) ABTS free radical scavenging capacity. (**c**) Hydroxyl radical scavenging capacity. (**d**) Superoxide anion radical scavenging capacity. (**e**) Level of ROS in U251MG cells after treatment with raw and cooked onion extracts. * *p* < 0.05, ** *p* < 0.01, *** *p* < 0.005, ### *p* < 0.001. The results are shown as the mean ± SD (*n* = 6).

DPPH analysis is suitable for hydrophobic compounds, whereas ABTS is suitable for both hydrophilic and hydrophobic compounds, providing a flexible reference for evaluating the antioxidant activity of samples in different aspects [49,50]. In the range of 0.05–100 mg/mL, raw and cooked onion extracts showed the same trend of scavenging DPPH free radicals, and the scavenging ability was gradually enhanced with the increase in concentration. Figure 7a shows that there was no significant difference in the DPPH scavenging capacity of raw and cooked onions at low concentrations. Interestingly, cooked onion extracts scavenged DPPH free radicals better than raw onion extracts, showing significant differences at concentrations of 50, 5, and 1 mg/mL. As shown in Figure 7b, onion extracts could scavenge ABTS radicals in a concentration-dependent manner. From the results, it showed the same phenomenon as DPPH. At high concentrations, the scavenging of ABTS radicals by the two onion extracts was saturated and not significantly different, whereas at low concentrations, the two onion extracts exhibited equally low scavenging capacity. We observed that the ABTS radical scavenging ability of cooked onion extracts was superior to that of raw onions in the medium concentration range (5–25 mg/mL), with a significant difference. It was found that too high temperature and pressure would decrease the free radical scavenging ability. The ability of onion extract to scavenge DPPH free radicals at 125 °C, 0.15 MPa is demonstrated in Supplementary Materials Table S1.

The ability of raw and cooked onion extracts to scavenge hydroxyl radicals and superoxide anion radicals was assessed, with the results presented in Figure 7c,d. Raw and cooked onion extracts can scavenge hydroxyl radicals and superoxide anion radicals in the 0.1–100 mg/mL concentration range. At concentrations below 0.1 mg/mL, onions no longer exhibit scavenging ability. As can be seen from the graph, the scavenging ability of both free radicals was better for cooked onion extracts than for raw onion extracts. However, cooked onion extracts did not show a significant advantage for superoxide anion radicals, whereas in terms of hydroxyl radical scavenging ability, cooked onion extracts were significantly better than raw onion extracts at 10 and 50 mg/mL. This disparity in antioxidant capacity between raw and cooked onions has been mentioned in the studies of Nooshkam et al. [51], who concluded that the Maillard reaction increases the antioxidant capacity of foods.

Although onion extracts have a significant free radical scavenging ability in vitro, further testing is required to determine its ability to exert antioxidant function in complex cellular environments. Figure 7e shows that the intracellular ROS level was significantly increased in U251 cells after 24 h of injury by 10 μM lead(II) ions, which indicates that lead(II) ions cause cellular damage mainly through oxidative stress. It is consistent with previous reports [47,52]. After treatment with both onion extracts for 24 h, the intracellular level of ROS in the damaged cells was significantly reduced. Cooked onion extracts at 1000 μg/mL reduced the level of ROS in U251MG cells significantly better than raw onion extracts.

#### 3.2.4. Onions Protect Nerve Cells by Chelating Lead(II) Ions

**Figure 8:**
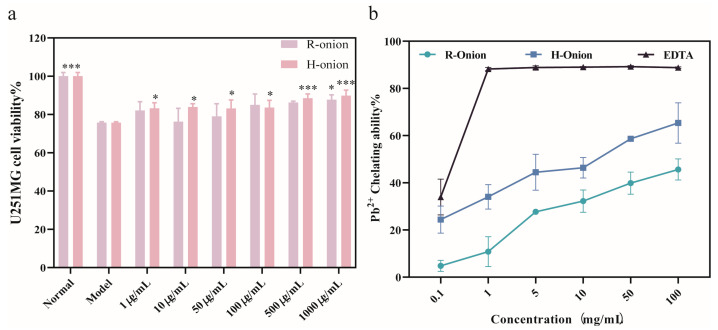
Cell viability and the ability of onions to chelate lead(II) ions. (**a**) Cell viability of U251MG cells stimulated simultaneously with 10 μM lead(II) ions and raw and cooked onion extracts for 48 h. (**b**) Raw onion extracts, cooked onion extracts, and EDTA chelating lead(II) ions capacity. * *p* < 0.05, *** *p* < 0.005. The results are shown as the mean ± SD (*n* = 6).

Multiple studies have demonstrated that sulfhydryl groups are prone to react with metal ions because of their distinctive structure. This can be attributed to two factors: first, the extra-nuclear electrons of the sulfur atom are highly polarizable, and second, the d-orbital electrons of the sulfur atom are predisposed to form hybridized orbitals with other electrons [53,54]. Disulfides and polysulfides can be converted to sulfhydryl groups in living organisms, at high temperatures, or in the presence of reducing agents [55]. Onions are rich in various sulfur-containing compounds. Two experiments were conducted to investigate whether onion compounds could have a protective effect on nerve cells by chelating lead(II) ions. First, we used the CCK-8 assay to investigate the changes in the viability of PC12 cells and U251MG cells in the presence of both lead(II) ions and onion extracts. The results are shown in Figure 8a. In U251MG cells, raw onion extracts significantly increased cell viability at a concentration of 1000 μg/mL, whereas cooked onion extracts increased cell viability at concentrations ranging from 1 to 1000 μg/mL, with the effect decreasing with decreasing concentration. This phenomenon may suggest that the lead ions react with certain components in the onion extract prior to damaging the cells, causing a change in the lead and thereby reducing its detrimental effects on the cells. The results of PC12 cells are shown in Figure S3.

In addition, we investigated whether onion extracts could chelate lead(II) ions through in vitro experiments using spectrophotometry, with EDTA as a positive control. As shown in Figure 8b, the values of the chelating capacity of raw onions for lead ions ranged from 5% to 50% over the range of concentrations set for the experiment (0.1–100 mg/mL), whereas the chelating capacity of cooked onions ranged from 20% to 75%. Both chelating capacities decreased with decreasing concentration. The chelating ability of cooked onion extracts is significantly better than that of raw onion extracts, which may be attributed to the occurrence of the Maillard reaction during the cooking process, which enhances the metal chelating ability of the food [51].

## 4. Discussion

Onions are easy to store and nutritious, and raw and cooked processing are the two main means of processing onions. Cooking brings many changes to the onion, increasing the content of flavorful substances (e.g., certain amino acids and nucleosides) and changing the onion’s flavor. The increase in nucleosides may be due to the high temperature and pressure during cooking, which causes DNA fragmentation within plant cells, producing nucleosides and other DNA degradation products [22,23,54,56]. Changes in several compounds may lead to differences in physiological function between raw and cooked onions. The present study offers guidance for various populations on whether to consume onions raw or cooked, and it supports the quality control of onions during food processing.

We found that raw and cooked onion extracts increased cell survival and viability after lead(II) ions damaged nerve cells. It is a fact that nerve cells are highly specialized. However, it is also a fact that many drugs fall short of protecting them. Because they are unable to cross the blood–brain barrier, it is crucial that we find solutions to this challenge to safeguard our nervous system and overall health. As a natural source of flavonoids and organosulfur compounds, onions have the unique advantage of protecting nerve cells. Our experimental results indicate that quercetin glucoside conjugates (quercetin-3β-D-glucoside) content, which is the most bioavailable in humans, increases after cooking. It has been reported that the content of flavonoids and organosulfur compounds in onion extracts can increase slightly or remain stable in a colonic fermentation model [57]. Flavonoids have been found to cross the blood–brain barrier [58]. Some studies have demonstrated that organosulfur compounds have therapeutic or protective effects against neurological disorders in rats [59]. Garlic extracts have similarities with onion extracts, and garlic extracts (FruArg) cross the blood–brain barrier [60]. These results imply that onion extracts are promising protective agents that may protect nerve cells from lead(II) ion damage.

Cooking enhances the onion extract’s ability to scavenge free radicals and chelate lead(II) ions. This is likely related to the Maillard reaction. Maillard reaction products (MRPs) are widely generated in foods containing reducing sugars and amino-bearing compounds during thermal processing and storage. Typically, these MRPs exert their antioxidant functions through mechanisms such as scavenging free radicals, interrupting radical chain reactions, chelating metal ions, reducing electrons, scavenging reactive oxygen species, and decomposing peroxides [51,61]. This study provides a theoretical reference for further development of safe and reliable antidote reagents for lead poisoning.

## 5. Conclusions

In summary, in this study, certain amino acids (e.g., DL-glutamine and L-pyroglutamic acid) in onions were found to undergo changes in content due to cooking. The levels of DNA fragmentation products, including adenine and N6-(2-furylmethyl)-9H-purin-6-amine, were observed to increase after cooking. Additionally, this study demonstrated that onion extracts possess protective effects against lead-induced neurotoxicity. Furthermore, cooking increases the antioxidant activity and lead-chelating capacity of onion extracts.

## Data Availability

The original contributions presented in the study are included in the article/Supplementary Materials; further inquiries can be directed to the corresponding author.

## Supplementary Materials

The following supporting information can be downloaded at: https://www.mdpi.com/article/10.3390/foods13223707/s1, A: Figure S1: Cell viability of PC12 cells; Figure S2: Cell viability and cell morphology of PC12 cells; Figure S3: Cell viability of PC12 cells stimulated simultaneously with 100 μM lead(II) ions and raw and cooked onion extracts for 48 h; Table S1: DPPH free radical scavenging capacity. B: Table S2: Information of 3136 positive ion signals. Table S3: Information of 1162 negative ion signals. Table S4: Information on 143 compounds with best match score higher than 80% in the positive ion mode. Table S5: Information on 59 compounds with best match score higher than 80% in the negative ion mode.

## Abbreviations

UHPLC–MS: ultrahigh-resolution liquid chromatograph–mass spectrometer; CCK–8: cell counting kit-8; DCFH–DA: dichlorofluorescin diacetate; DPPH: 1 1,1-diphenyl-2-picrylhydrazyl; ABTS: 2,2′-azino-bis(3-ethylbenzothiazoline-6-sulfonic acid); PAR: 4-(2-pyridylazo)resorcinol; GC–MS: Gas chromatography–mass spectrometry; AGEs: advanced glycation end products; SMC: S-methyl-L-cystein; SAC: S-allyl-L-cysteine; SMCA: S-methyl-L-cysteine sulfoxide; DADS: diallyl disulfide; DAS: diallyl sulfide; MRPs: Maillard reaction products.

## References

[1] Abdelrahman M., Ariyanti N.A., Sawada Y., Tsuji F., Hirata S., Hang T.T.M., Okamoto M., Yamada Y., Tsugawa H., Hirai M.Y. (2020). Metabolome-Based Discrimination Analysis of Shallot Landraces and Bulb Onion Cultivars Associated with Differences in the Amino Acid and Flavonoid Profiles. Molecules.

[2] Teshika J.D., Zakariyyah A.M., Zaynab T., Zengin G., Rengasamy K.R., Pandian S.K., Fawzi M.M. (2019). Traditional and modern uses of onion bulb (*Allium cepa* L.): A systematic review. Crit. Rev. Food Sci. Nutr..

[3] Moreno-Rojas J.M., Moreno-Ortega A., Ordóñez J.L., Moreno-Rojas R., Pérez-Aparicio J., Pereira-Caro G. (2018). Development and validation of UHPLC-HRMS methodology for the determination of flavonoids, amino acids and organosulfur compounds in black onion, a novel derived product from fresh shallot onions (*Allium cepa* var. aggregatum). Lwt-Food Sci. Technol..

[4] Li J., Dadmohammadi Y., Abbaspourrad A. (2021). Flavor components, precursors, formation mechanisms, production and characterization methods: Garlic, onion, and chili pepper flavors. Crit. Rev. Food Sci. Nutr..

[5] Williams R.J., Holladay S.D., Williams S.M., Gogal R.M. (2018). Environmental Lead and Wild Birds: A Review. Rev. Environ. Contam. Toxicol..

[6] Harshitha P., Bose K., Dsouza H.S. (2024). Influence of lead-induced toxicity on the inflammatory cytokines. Toxicology.

[7] van Wijngaarden E., Dosemeci M. (2006). Brain cancer mortality and potential occupational exposure to lead: Findings from the National Longitudinal Mortality Study, 1979–1989. Int. J. Cancer.

[8] Cai S., Liu J., Shi X., Hu S., Zhao L. (2019). Allicin alleviated learning and memory deficits caused by lead exposure at developmental stage. Life Sci..

[9] Chen L.J., Yang X.Q., Jiao H.L., Zhao B.L. (2002). Tea catechins protect against lead-induced cytotoxicity, lipid peroxidation, and membrane fluidity in HepG2 cells. Toxicol. Sci..

[10] Shi Y., Tian C., Yu X., Fang Y., Zhao X., Zhang X., Xia D. (2020). Protective Effects of Smilax glabra Roxb. Against Lead-Induced Renal Oxidative Stress, Inflammation and Apoptosis in Weaning Rats and HEK-293 Cells. Front. Pharmacol..

[11] Baran E.J. (2010). Chelation therapies: A chemical and biochemical perspective. Curr. Med. Chem..

[12] Böttcher C., Krähmer A., Stürtz M., Widder S., Schulz H. (2017). Comprehensive metabolite profiling of onion bulbs (*Allium cepa*) using liquid chromatography coupled with electrospray ionization quadrupole time-of-flight mass spectrometry. Metabolomics.

[13] Jiang S., Shi F., Lin H., Ying Y., Luo L., Huang D., Luo Z. (2020). Inonotus obliquus polysaccharides induces apoptosis of lung cancer cells and alters energy metabolism via the LKB1/AMPK axis. Int. J. Biol. Macromol..

[14] Liu M.-X., Jin L., Sun S.-J., Liu P., Feng X., Cheng Z.-L., Liu W.-R., Guan K.-L., Shi Y.-H., Yuan H.-X. (2018). Metabolic reprogramming by PCK1 promotes TCA cataplerosis, oxidative stress and apoptosis in liver cancer cells and suppresses hepatocellular carcinoma. Oncogene.

[15] Jing L., Ma H., Fan P., Gao R., Jia Z. (2015). Antioxidant potential, total phenolic and total flavonoid contents of Rhododendron anthopogonoides and its protective effect on hypoxia-induced injury in PC12 cells. BMC Complement. Altern. Med..

[16] Hong J., Hu J.-Y., Liu J.-H., Zhou Z., Zhao A.-F. (2014). In vitro antioxidant and antimicrobial activities of flavonoids from *Panax notoginseng* flowers. Nat. Prod. Res..

[17] Low S.C., Azmi N.A.b., Ong C.S., Lim J.K. (2022). Environmental monitoring of trace metal pollutants using cellulosic-paper incorporating color change of azo-chromophore. Environ. Sci. Pollut..

[18] Yuejuan F., Daozong X. (2016). Study on Chelating Ability of Natural Product Extracts to Lead Ions by Spectrophotometric Method. Chin. Arch. Tradit. Chin. Med..

[19] Barciszewski J., Massino F., Clark B.F.C. (2007). Kinetin—A multiactive molecule. Int. J. Biol. Macromol..

[20] Miller C.O., Skoog F., Okumura F.S., Von Saltza M.H., Strong F.M. (1956). Isolation, Structure and Synthesis of Kinetin, a Substance Promoting Cell Division1,2. J. Am. Chem. Soc..

[21] Kadlecová A., Maková B., Artal-Sanz M., Strnad M., Voller J. (2019). The plant hormone kinetin in disease therapy and healthy aging. Ageing Res. Rev..

[22] De Angelis M., Gobbetti M. (2004). Environmental stress responses in *Lactobacillus*: A review. Proteomics.

[23] Dizdaroglu M., Bergtold D.S. (1986). Characterization of free radical-induced base damage in DNA at biologically relevant levels. Anal. Biochem..

[24] Park Y., Peoples A.R., Madugundu G.S., Sanche L., Wagner J.R. (2013). Side-by-Side Comparison of DNA Damage Induced by Low-Energy Electrons and High-Energy Photons with Solid TpTpT Trinucleotide. J. Phys. Chem. B.

[25] Comunian T.A., Grassmann Roschel G., da Silva Anthero A.G., de Castro I.A., Dupas Hubinger M. (2020). Influence of heated, unheated whey protein isolate and its combination with modified starch on improvement of encapsulated pomegranate seed oil oxidative stability. Food Chem..

[26] Wei H., Zhou H., Huang L., Zhang Q., Teng J., Wei B., Xia N., Qin R., Zhu P. (2023). Effect of baking treatment on quality, nonvolatile and volatile compounds of Liupao tea. Int. J. Food Sci. Technol..

[27] Coleman S., Linderman R., Hodgson E., Rose R.L. (2000). Comparative metabolism of chloroacetamide herbicides and selected metabolites in human and rat liver microsomes. Environ. Health Perspect..

[28] Gazme B., Boachie R.T., Tsopmo A., Udenigwe C.C. (2019). Occurrence, properties and biological significance of pyroglutamyl peptides derived from different food sources. Food Sci. Hum. Well.

[29] Feng Y.-H., Zhang G.-D., Zhu P.-C., Zhu W.-H., Li Y.-Z., Fan X.-W. (2023). Metabolite profiles and antibacterial and antioxidant activities of leaf extracts of five *Lonicera* species: A comparative study. Chem. Biol. Technol. Agric..

[30] He X., Yang Y., Yuan X., Sun Y., Li Y. (2023). Chemical composition and anticonvulsant activities of herb pair of *Gastrodia elata* Blume-Acorus tatarinowii Schott decoction on experimentally induced seizures in mice. Metab. Brain Dis..

[31] Bouzekri O., Elgamouz S., Ghaleb A., Amechrouq A., El Idrissi M., Choukrad M. (2022). Valorization of perillaldehyde molecule contained in the essential oil of *Ammodaucus leucotrichus* Coss. from the Saharan zones of Morocco. J. Microbiol..

[32] Jia X., An Q., Zhang N., Ren J., Pan S., Zheng C., Zhou Q., Fan G. (2024). Recent advances in the contribution of glucosinolates degradation products to cruciferous foods odor: Factors that influence degradation pathways and odor attributes. Crit. Rev. Food Sci. Nutr..

[33] Kondjoyan A., Portanguen S., Duchène C., Mirade P.S., Gandemer G. (2018). Predicting the loss of vitamins B3 (niacin) and B6 (pyridoxamine) in beef during cooking. J. Food Eng..

[34] MacKay D., Hathcock J., Guarneri E. (2012). Niacin: Chemical forms, bioavailability, and health effects. Nutr. Rev..

[35] Cai H., Shen Y. (2024). Metabolomic and Physiological Analyses Reveal the Effects of Different Storage Conditions on *Sinojackia xylocarpa* Hu Seeds. Metabolites.

[36] Kung P.-P., Zehnder L.R., Meng J.J., Kupchinsky S.W., Skalitzky D.J., Johnson M.C., Maegley K.A., Ekker A., Kuhn L.A., Rose P.W. (2005). Design, synthesis, and biological evaluation of novel human 5′-deoxy-5′-methylthioadenosine phosphorylase (MTAP) substrates. Bioorganic Med. Chem. Lett..

[37] Yao H., Xu Y.L., Liu W., Lu Y., Gan J.H., Liu Y., Tao N.P., Wang X.C., Xu C.H. (2021). Taste compounds generation and variation of broth in pork meat braised processing by chemical analysis and an electronic tongue system. J. Food Biochem..

[38] Le H.T.N., Van Roy E., Dendooven E., Peeters L., Theunis M., Foubert K., Pieters L., Tuenter E. (2021). Alkaloids from *Lepidium meyenii* (Maca), structural revision of macaridine and UPLC-MS/MS feature-based molecular networking. Phytochemistry.

[39] Li X., Yang Z., Deng J., Chen C., Xu B., Li P. (2023). Effect of quercetin and oil water separation system on formation of β-carboline heterocyclic amines during frying process of braised chicken drumsticks. Curr. Res. Food Sci..

[40] Chang C.-C., Kao T.-H., Zhang D., Wang Z., Inbaraj B.S., Hsu K.-Y., Chen B.H. (2018). Application of QuEChERS Coupled with HPLC-DAD-ESI-MS/MS for Determination of Heterocyclic Amines in Commercial Meat Products. Food Anal. Methods.

[41] Dabeek W.M., Marra M.V. (2019). Dietary Quercetin and Kaempferol: Bioavailability and Potential Cardiovascular-Related Bioactivity in Humans. Nutrients.

[42] de Vries J.H., Hollman P.C., Meyboom S., Buysman M.N., Zock P.L., van Staveren W.A., Katan M.B. (1998). Plasma concentrations and urinary excretion of the antioxidant flavonols quercetin and kaempferol as biomarkers for dietary intake. Am. J. Clin. Nutr..

[43] de Vries J.H., Hollman P.C., van Amersfoort I., Olthof M.R., Katan M.B. (2001). Red wine is a poor source of bioavailable flavonols in men. J. Nutr..

[44] Olthof M.R., Hollman P.C., Vree T.B., Katan M.B. (2000). Bioavailabilities of quercetin-3-glucoside and quercetin-4′-glucoside do not differ in humans. J. Nutr..

[45] Hollman P.C., van Trijp J.M., Buysman M.N., van der Gaag M.S., Mengelers M.J., de Vries J.H., Katan M.B. (1997). Relative bioavailability of the antioxidant flavonoid quercetin from various foods in man. FEBS Lett..

[46] Adan A., Kiraz Y., Baran Y. (2016). Cell Proliferation and Cytotoxicity Assays. Curr. Pharm. Biotechnol..

[47] Guo Y., Ma H., Huang Q. (2022). Yeast β-glucan with different degrees of oxidation: Capability of adsorbing lead ions and protective effect against lead-induced PC12 cytotoxicity. Int. J. Biol. Macromol..

[48] Li N., Wen L., Li T., Yang H., Qiao M., Wang T., Song L., Huang X., Li M., Bukyei E. (2022). Alleviating Effects of Black Soybean Peptide on Oxidative Stress Injury Induced by Lead in PC12 Cells via Keap1/Nrf2/TXNIP Signaling Pathway. Nutrients.

[49] Metrani R., Singh J., Acharya P., K. Jayaprakasha G., S. Patil B. (2020). Comparative Metabolomics Profiling of Polyphenols, Nutrients and Antioxidant Activities of Two Red Onion (*Allium cepa* L.) Cultivars. Plants.

[50] Schaich K.M., Tian X., Xie J. (2015). Hurdles and pitfalls in measuring antioxidant efficacy: A critical evaluation of ABTS, DPPH, and ORAC assays. J. Funct. Foods.

[51] Nooshkam M., Varidi M., Bashash M. (2019). The Maillard reaction products as food-born antioxidant and antibrowning agents in model and real food systems. Food Chem..

[52] Flora G., Gupta D., Tiwari A. (2012). Toxicity of lead: A review with recent updates. Interdiscip. Toxicol..

[53] Bjørklund G., Crisponi G., Nurchi V.M., Cappai R., Buha Djordjevic A., Aaseth J. (2019). A Review on Coordination Properties of Thiol-Containing Chelating Agents Towards Mercury, Cadmium, and Lead. Molecules.

[54] Tandon S.K., Sharma B.L., Singh S. (1988). Chelation in Metal Intoxication XXVII: Chelating Agents Containing Vicinal Thioether Groups as Antidotes of Lead Toxicity. Drug Chem. Toxicol..

[55] Iciek M., Kowalczyk-Pachel D., Bilska-Wilkosz A., Kwiecień I., Górny M., Włodek L. (2016). S-sulfhydration as a cellular redox regulation. Biosci. Rep..

[56] Feng Y., Fan X., Zhang S., Yu M., Wu T., Liang Y., Wang C., Yang H. (2022). Effect of Thermal Processing on the Metabolic Components of Black Beans on Ultra-High-Performance Liquid Chromatography Coupled with High-Field Quadrupole-Orbitrap High-Resolution Mass Spectrometry. Molecules.

[57] Moreno-Ortega A., Di Pede G., Mena P., Calani L., Del Rio D., Moreno-Rojas J.M., Pereira-Caro G. (2022). Effects of colonic fermentation on the stability of fresh and black onion bioactives. Food Funct..

[58] Deepika, Maurya P.K. (2022). Health Benefits of Quercetin in Age-Related Diseases. Molecules.

[59] Hegazy E., Sabry A., Khalil W.K.B. (2022). Neuroprotective effects of onion and garlic root extracts against Alzheimer’s disease in rats: Antimicrobial, histopathological, and molecular studies. BioTechnologia.

[60] Song H., Cui J., Mossine V., Greenlief C., Fritsche K., Sun G., Gu Z. (2019). Bioactive components from garlic on brain resiliency against neuroinflammation and neurodegeneration (Review). Exp. Ther. Med..

[61] Shakoor A., Zhang C., Xie J., Yang X. (2022). Maillard reaction chemistry in formation of critical intermediates and flavour compounds and their antioxidant properties. Food Chem..

