# Supplementary material for: Cooking Alters the Metabolites of Onions and Their Ability to Protect Nerve Cells from Lead Damage
Source: Foods. 2024 Nov 20;13(22):3707. doi: 10.3390/foods13223707 (PMC11593875; doi:10.3390/foods13223707)
Supplement: Supplementary file 1 [file foods-13-03707-s001.zip › Supplementary Material-A.pdf]

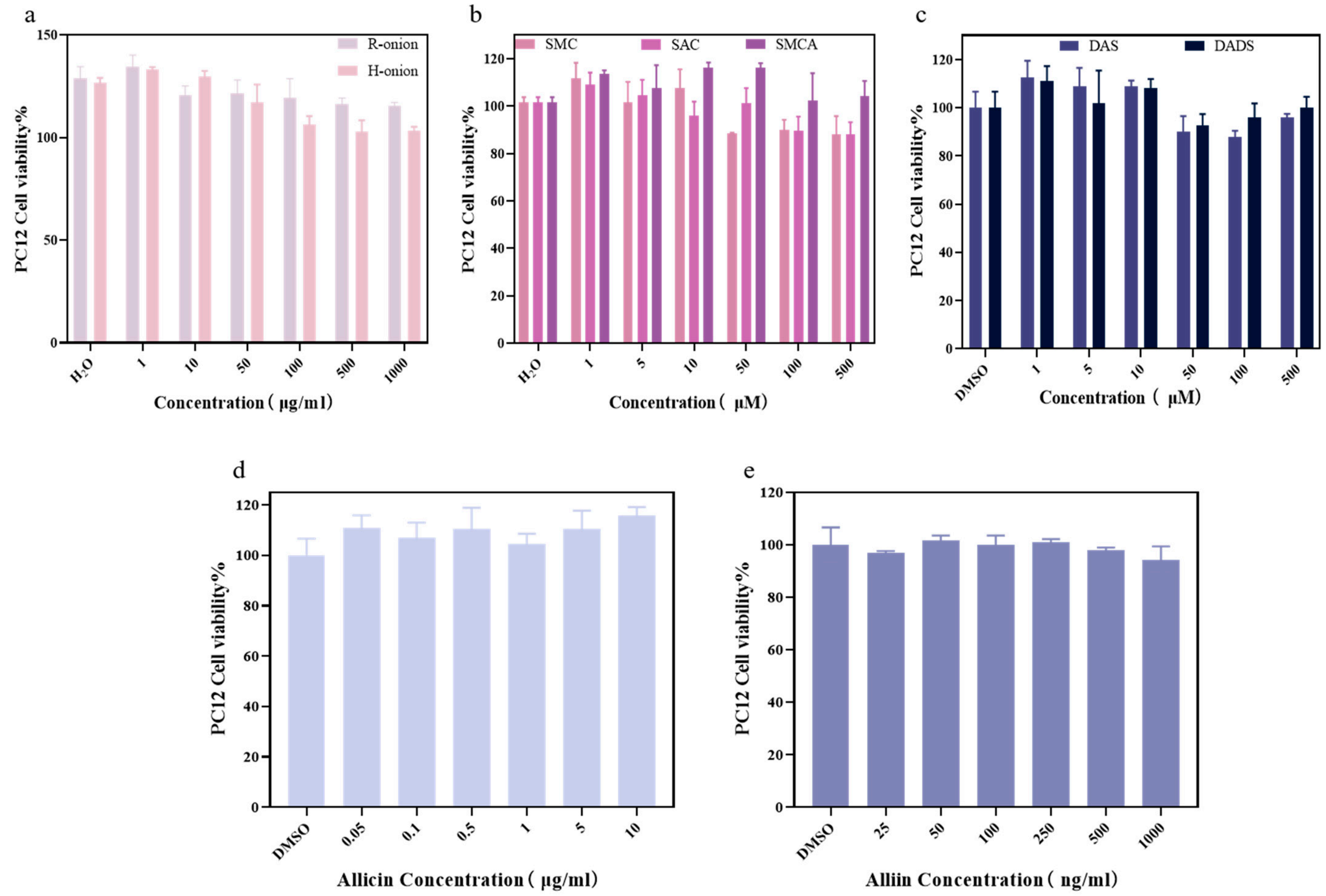

Figure S1. Cell viability of PC12 cells. (a). Cell viability of PC12 treated with raw onion extract and cooked onion extract for 48 h; (b). Cell viability of PC12 treated with SMC, SAC, and SMCA for 48 h; (c). Cell viability of PC12 treated with DAS and DADS for 48 h; (d). Cell viability of PC12 treated with Allicin for 48 h; (e). Cell viability of PC12 treated with Alliin for 48 h. ** $P < 0.01$, *** $P < 0.001$. The results were shown as the mean ± SD (n = at least 3)

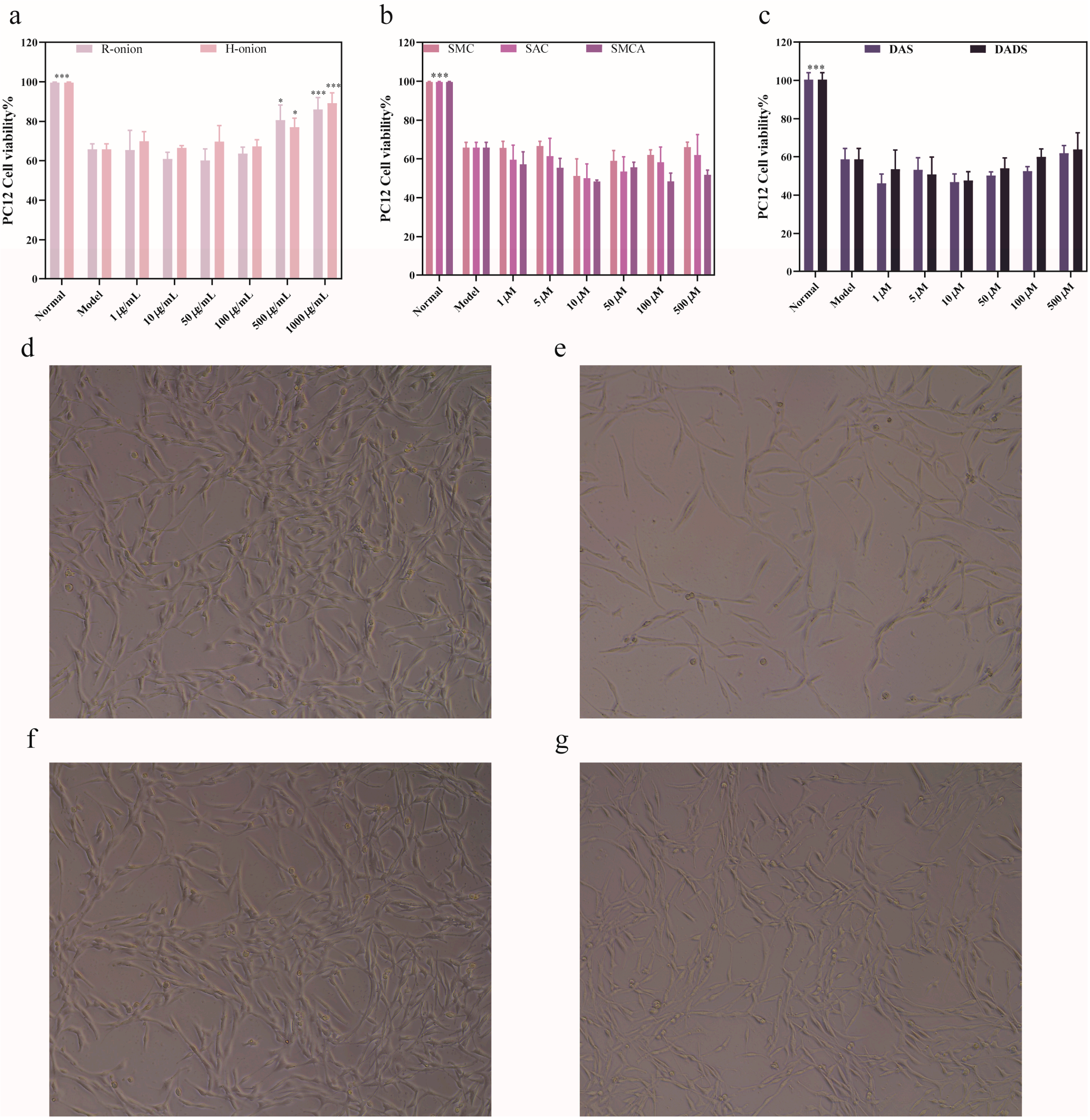

Figure S2. Cell viability and cell morphology of PC12 cells. (a). Cell viability of 100 μM lead(II)-injured cells for 24 h followed by treatment of cells with raw onion extract and cooked onion extract for 24 h; (b). Cell viability of 10 μM lead(II)-injured cells for 24 h followed by treatment of cells with SMC, SAC, and SMCA for 24 h; (c). Cell viability of 10 μM lead(II)-injured cells for 24 h followed by treatment of cells with DAS and DADS for 24 h; (d). Cell morphology of PC12 cells in the normal group; (e). Cell morphology of PC12 cells in the model group; (f). Cell morphology of PC12 cells in raw onion 1000 μg/mL group; (g). Cell

morphology of PC12 cells in cooked onion 1000 μg/mL group. * $P < 0.05$, ** $P < 0.01$, *** $P < 0.001$. The results were shown as the mean ± SD (n = at least 3)

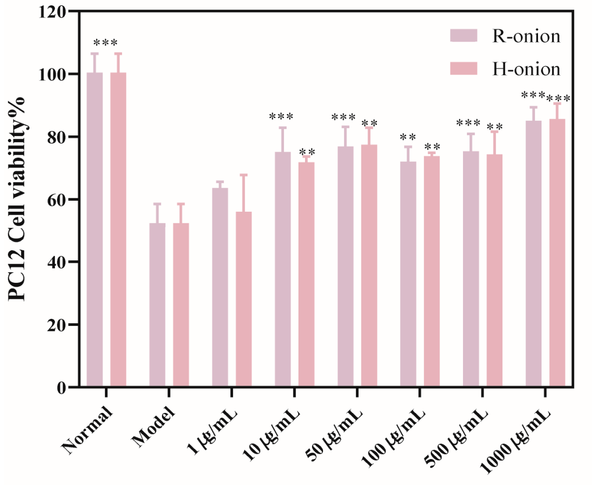

Figure S3. Cell viability of PC12 cells stimulated simultaneously with 100 μM lead(II) ions and raw and cooked onion extracts for 48 h. ** $P < 0.01$, *** $P < 0.001$. The results were shown as the mean ± SD (n = at least 3)

Table S1. DPPH free radical scavenging capacity

Concentration (mg/ml)	Free radical scavenging capacity (%)		
	R-onion	H-onion	Hp-onion（125℃, 0.15MPa）
0.01	16.78±3.60	13.58±5.90	7.94±1.40
0.05	17.11±6.84	13.02±6.33	18.09±4.61
0.1	18.54±2.51	16.14±2.35	20.15±7.77
0.5	26.91±0.56	33.87±1.99	21.80±13.02
1	32.43±2.23	43.96±1.32	44.08±5.73
1.5	38.36±0.79	53.48±4.59	43.26±4.58

The results were shown as the mean ± SD (n = 3)
